# Results of Two Years Expenience with Fluorescence Bronchoscopy in Detection of Preinvasive Bronchial Neoplasia

**DOI:** 10.1155/DTE.5.77

**Published:** 1999

**Authors:** Ben J. W. Venmans, Ton J. M. Van Boxem, Egbert F. Smit, Pieter E. Postmus, Tom G. Sutedja

**Affiliations:** 1 Department of Pulmonary Medicine University Hospital Vrije Universiteit De Boelelaan 1117 Amsterdam 1081HV The Netherlands; 2 Department of Pulmonary Medicine University Hospital Vrije Universiteit PO Box 7057 Amsterdam 1007 MB The Netherlands

## Abstract

The aim of the study was to determine whether use of fluorescence bronchoscopy improves the detection of preinvasive neoplastic bronchial lesions. The data of all patients who underwent fluorescence bronchoscopy and in whom bronchial biopsies were taken, were analyzed. Most patients were at risk for preinvasive lesions. A total of 174 bronchoscopies were performed in 95 patients. Of the 681 representative biopsies, 31 were found to be moderate dysplastic, 39 were found to be severe dysplastic and 9 exhibited carcinoma *in situ.*  These 79 preinvasive lesions were found in 34 patients. The respective results of fluorescence bronchoscopy in addition to conventional bronchoscopy and of conventional bronchoscopy alone for detection of preinvasive lesions were: sensitivity 85% (67/79) and 59% (47/79); specificity 60% (351/581) and 85% (493/581); positive predictive values of 23% (67/297) and 35% (47/135); negative predictive values of 97% (351/363) and 94% (493/525). A separate analysis of only the first bronchoscopy of each patient showed similar results. Results of fluoresence bronchoscopy were better in the second part of the patient group. We conclude that after a learning period fluorescence bronchoscopy can increase the yield of finding preinvasive neoplastic lesions when used in addition to conventional bronchoscopy.


*Diagnostic and Therapeutic Endoscopy*, Vol. 5, pp. 77–84
Reprints available directly from the publisher
Photocopying permitted by license only

© 1999 OPA (Overseas Publishers Association) N.V.
Published by license under
the Harwood Academic Publishers imprint,
part of The Gordon and Breach Publishing Group.
Printed in Singapore.

### Results of Two Years Experience with Fluorescence Bronchoscopy in Detection of Preinvasive Bronchial Neoplasia

BEN J.W. VENMANS, TON J.M. VAN BOXEM, EGBERT F. SMIT, PIETER E. POSTMUS and TOM G. SUTEDJA*

*Department of Pulmonary Medicine, University Hospital Vrije Universiteit, De Boelelaan 1117, 1081HV Amsterdam, The Netherlands*

**The aim of the study was to determine whether use of fluorescence bronchoscopy improves the detection of preinvasive neoplastic bronchial lesions. The data of all patients who underwent fluorescence bronchoscopy and in whom bronchial biopsies were taken, were analyzed. Most patients were at risk for preinvasive lesions. A total of 174 bronchoscopies were performed in 95 patients. Of the 681 representative biopsies, 31 were found to be moderate dysplastic, 39 were found to be severe dysplastic and 9 exhibited carcinoma *in situ*. These 79 preinvasive lesions were found in 34 patients. The respective results of fluorescence bronchoscopy in addition to conventional bronchoscopy and of conventional bronchoscopy alone for detection of preinvasive lesions were: sensitivity 85% (67/79) and 59% (47/79); specificity 60% (351/581) and 85% (493/581); positive predictive values of 23% (67/297) and 35% (47/135); negative predictive values of 97% (351/363) and 94% (493/525). A separate analysis of only the first bronchoscopy of each patient showed similar results. Results of fluoresence bronchoscopy were better in the second part of the patient group. We conclude that after a learning period fluorescence bronchoscopy can increase the yield of finding preinvasive neoplastic lesions when used in addition to conventional bronchoscopy.**

*Keywords:* Early lung cancer, Fluorescence bronchoscopy, Intraepithelial neoplasia, LIFE, Lung imaging fluorescence endoscope, Preinvasive neoplasia

### INTRODUCTION

Squamous cell carcinoma of the bronchus is thought to arise after a series of progressive histologic changes in the bronchial epithelium [1]. These preinvasive neoplastic lesions are, in order of increasing severity: basal cell hyperplasia, metaplasia, mild, moderate and severe dysplasia, and

* Corresponding author. Department of Pulmonary Medicine, University Hospital Vrije Universiteit, PO Box 7057, 1007 MB Amsterdam, The Netherlands. Tel.: 31-20-444 4782. Fax:31-20-444 4328. E-mail: tg.sutedja@azvu.nl.

carcinoma *in situ*. When the bronchial mucosa is illuminated by a blue light with a wavelength of 405–442 nm, such as light from a Krypton ion laser or a Helium–Cadmium laser, there is a progressive reduction in the green wavelength band of the autofluorescence spectrum as the tissue becomes more neoplastic [2,3]. Based on this difference in fluorescence intensity, the lung imaging fluorescence endoscope (LIFE) system (Xillix Technologies Corp; Richmond, BC, Canada) was developed to detect preinvasive bronchial lesions [4,5]. LIFE provides true fluorescence images in real time. Normal tissue which fluoresces green, can be distinguished from neoplastic tissue which appears to be brown or brownish red in color depending on the pathology grade. Experience with the LIFE system has shown that it can improve the detection rate of preinvasive bronchial lesions compared with conventional bronchoscopy. Lam *et al.* [5] found the sensitivity of LIFE to be twice the sensitivity of conventional bronchoscopy for the detection of preinvasive bronchial lesions. Fluorescence bronchoscopy could therefore be clinically useful in detecting and localizing early lung cancer, in staging the extent of endobronchial spread of bronchial cancer and in detecting synchronous roentgenographically occult cancer. Early detection may improve the prognosis of lung cancer patients, since it was shown that early treatment as surgical resection or bronchoscopic treatment for roentgenographically occult intraluminal tumors gives the best chance for cure [6–14]. We started using the LIFE system in clinical practice in November 1995. The aim of this study was to determine whether use of the LIFE system improves the detection of preinvasive bronchial lesions. Preinvasive lesions were defined as lesions that were scored by the pathologist as moderate dysplasia, severe dysplasia or carcinoma *in situ*. These preinvasive lesions were considered clinically important in the population at risk, and were considered to be the interest of using a LIFE system for early tumor detection.

### MATERIALS AND METHODS

### Bronchoscopy

All the patients from the Department of Pulmonary Medicine of the University Hospital Vrije Universiteit in Amsterdam who underwent a LIFE bronchoscopy in the period from November 1995 to December 1997 and in whom bronchial biopsies were taken were included in this study. The results of 33 of these patients were reported before [15]. In these 33 patients the modality of the initial bronchoscopy to be performed, conventional bronchoscopy or LIFE, was detemined by randomization. Indications for LIFE bronchoscopy in most patients of the present study were: suspected lung cancer, staging of newly diagnosed lung cancer or follow-up of lung or head and neck cancer. All bronchoscopic procedures were performed by the same bronchoscopist (TS). Standard bronchoscopy procedure was followed. Local anesthesia with lidocaine 2% spraying was administered to all patients. In the decubitus position, the bronchoscope (Olympus BF-20D) was introduced transorally. The fiberoptic bronchoscope was coupled to either the conventional or the LIFE video camera and bronchoscopy was performed. Careful bronchoscopy is obligatory to prevent unnecessary mucosal contact, as hemorrhagic bruising may undermine imaging. Digitized images were videotaped and frozen images of abnormal or suspicious lesions were captured. All locations were documented. After switching the camera, bronchoscopic examination was repeated in the same manner using the alternate device. All abnormal and suspicious lesions were separately biopsied, using a new biopsy forceps for each location to prevent contamination. In all cases at least one biopsy was taken of an area considered not suspicious during both LIFE and conventional bronchoscopy. Abnormalities detected by the LIFE were biopsied using the LIFE camera to ensure the exact spot, unless images were too blurred because of hemorrhagic bruising of the mucosa during manipulation. Abnormalities detected only during conventional bronchoscopy were subsequently biopsied.

### Histological Examination

The biopsies were processed in a routine fashion. After fixation for at least 24 h in 4% buffered formalin, the tissue was blocked in paraffin and 4 µm slides were mounted on glass and stained with hematoxylin and eosin using standard procedure. The pathologists reported the results of microscopy of all specimens in separate jars, by indicating them to be: unsatisfactory, normal, inflammation, hyperplasia, mild dysplasia, moderate dysplasia, severe dysplasia, carcinoma *in situ*, and carcinoma.

### Analysis

The results of the histologic examination were compared with the results of the visual assessment using the conventional and the LIFE cameras. Sensitivity, specificity, positive and negative predictive values for detection of the total number of moderate dysplasias, severe dysplasias and carcinomas *in situ* were calculated. The relative sensitivity, or the ratio of the sensitivity of conventional bronchoscopy combined with LIFE, as compared with conventional bronchoscopy alone, along with the 95% confidence interval (CI), was calculated to evaluate the contribution of fluorescence examination to detect preinvasive lesions. The results of all bronchoscopies using the LIFE system were analyzed. In a subgroup of patients several LIFE bronchoscopies were done. Because patient selection may influence the results, a separate analysis was performed using only the first bronchoscopy data of each patient. This second analysis was also used to examine if there is a learning effect in using the LIFE system. We therefore compared the results of the first 48 patients with the results obtained in the next 47 consecutive patients.

### RESULTS

Patients demographics and bronchoscopy data are shown in Table I. A total of 174 bronchoscopies were performed in 95 patients. All procedures were completed under local anesthesia and no complications were observed during or after bronchoscopic examination. The median duration of the additional LIFE examination was 15 min (range: 5–25 min). Of the total number of biopsies taken, 92% (681/742) was judged by the pathologists to be satisfactory, enabling adequate assessment of the morphology. Three-hundred-and-two of these 681 biopsies were taken based on suspicious findings during LIFE bronchoscopy. One-hundred-and-fourty of these 302 biopsies were

TABLE I Patient demographics and bronchoscopy data

| | All bronchoscopies | First bronchoscopies |
|---|---|---|
| Number of patients | 95 | 95 |
| Male/female | 75/20 | 75/20 |
| Median age at bronchoscopy (range) | 65 (32–82) | 64 (32–82) |
| Total number of bronchoscopies | 174 | 95 |
| Median number of bronchoscopies (range) per patient | 1 (1–9) | 1 (1) |
| Indications for bronchoscopy (no. (%)) | | |
| Suspected or known lung cancer | 85 (49) | 64 (68) |
| Follow-up treated lung or head and neck cancer | 82 (47) | 25 (26) |
| Other | 7 (4) | 6 (6) |
| Total number of biopsies | 742 | 358 |
| Median number of biopsies per bronchoscopy (range) | 4 (1–11) | 3 (1–11) |

considered suspicious as well during conventional bronchoscopy. Sixteen biopsies were taken from areas considered suspicious during conventional bronchoscopy which were considered not suspicious by LIFE bronchoscopy. Overall 318 biopsies were taken from suspicious areas based on endoscopic findings (Table II). Of the 681 representative specimens, 31 were found to be moderate dysplastic, 39 were found to be severe dysplastic, 9 exhibited carcinoma *in situ* and 21 showed carcinoma. One moderate dysplasia, 2 severe dysplasias, 1 carcinoma *in situ* and 2 carcinomas were seen only using the conventional bronchoscope and were missed with the LIFE system. Eleven moderate dysplasias, 8 severe dysplasias and 1 carcinoma *in situ* were seen only using the LIFE. Twelve moderate dysplasias were missed by both modalities (Table II). The 79 preinvasive lesions were found in 34 patients (female/male 8/26) and the 21 carcinomas were found in 16 patients (female/male 2/14). A total of 12 preinvasive lesions were found in 6 of the latter 16 patients. The respective results of conventional bronchoscopy combined with LIFE and of conventional bronchoscopy alone for detection of the combined moderate dysplasias, severe dysplasias and carcinomas *in situ* were: sensitivity 85% (67/79) and 59% (47/79); specificity 60% (351/581) and 85% (493/581); positive predictive values of 23% (67/297) and 35% (47/135); negative predictive values of 97% (351/363) and 94% (493/525). These results are shown in Table IV. The relative sensitivity of conventional bronchoscopy combined with LIFE vs conventional bronchoscopy alone was 1.43 (95% CI 1.16–1.75).

A separate analysis of the results of only the first bronchoscopies showed that 94% (337/358) of the biopsies taken were judged to be satisfactory for histological examination. Fourty preinvasive lesions were found in 25 patients (female/male 5/20) and 16 carcinomas were found in 13 patients (female/male 1/12). A total of 3 preinvasive lesions were found in 3 of these 13 patients. Two of these 3 preinvasive lesions were found in other bronchi

TABLE II Data of all bronchoscopies and results of histologic examination

| Bronchoscopic assessment | No. of specimens | | | | |
|---|---|---|---|---|---|
| | Lesions satisfactory for histological examination | Moderate dysplasia | Severe dysplasia | Carcinoma *in situ* | Carcinoma |
| Suspicious by LIFE and CB | 140 | 7 | 29 | 7 | 19 |
| Suspicious by LIFE, not by CB | 162 | 11 | 8 | 1 | 0 |
| Suspicious by CB, not by LIFE | 16 | 1 | 2 | 1 | 2 |
| Not suspicious by LIFE and CB | 363 | 12 | 0 | 0 | 0 |
| Total | 681 | 31 | 39 | 9 | 21 |

CB = conventional bronchoscopy.

TABLE III Data of first bronchoscopies only and results of histologic examination

| Bronchoscopic assessment | No. of specimens | | | | |
|---|---|---|---|---|---|
| | Lesions satisfactory for histological examination | Moderate dysplasia | Severe dysplasia | Carcinoma *in situ* | Carcinoma |
| Suspicious by LIFE, and CB | 73 | 4 | 16 | 6 | 14 |
| Suspicious by LIFE, not by CB | 60 | 3 | 2 | 1 | 0 |
| Suspicious by CB, not by LIFE | 10 | 0 | 1 | 1 | 2 |
| Not suspicious by LIFE and CB | 194 | 6 | 0 | 0 | 0 |
| Total | 337 | 13 | 19 | 8 | 16 |

CB = conventional bronchoscopy.

TABLE IV Results of conventional bronchoscopy and LIFE and of the combined modalities in detecting preinvasive lesions.[a] All bronchoscopies included in analysis

| | CB | | LIFE | | CB/LIFE | |
|---|---|---|---|---|---|---|
| | % | No. | % | No. | % | No. |
| Sensitivity | 59 | 47/79 | 80 | 63/79 | 85 | 67/79 |
| Specificity | 85 | 493/581 | 62 | 361/581 | 60 | 351/581 |
| Positive predictive value | 35 | 47/135 | 22 | 63/283 | 23 | 67/297 |
| Negative predictive value | 94 | 493/525 | 96 | 361/377 | 97 | 351/363 |

[a]Biopsies showing cancer not included in analysis; CB = conventional bronchoscopy.

TABLE V Results of conventional bronchoscopy and LIFE and of the combined modalities in detecting preinvasive lesions.[a] Only first bronchoscopies included in analysis

| | CB | | LIFE | | CB/LIFE | |
|---|---|---|---|---|---|---|
| | % | No. | % | No. | % | No. |
| Sensitivity | 70 | 28/40 | 80 | 32/40 | 85 | 34/40 |
| Specificity | 86 | 242/281 | 69 | 194/281 | 67 | 188/281 |
| Positive predictive value | 42 | 28/67 | 27 | 32/119 | 27 | 34/127 |
| Negative predictive value | 95 | 242/254 | 96 | 194/202 | 97 | 188/194 |

[a]Biopsies showing cancer not included in analysis; CB = conventional bronchoscopy.

TABLE VI Results of LIFE bronchoscopy in detecting preinvasive lesions[a]

| | Patients 1–48 | | Patients 49–95 | |
|---|---|---|---|---|
| | % | No. | % | No. |
| Sensitivity | 67 | 8/12 | 86 | 24/28 |
| Specificity | 67 | 82/123 | 71 | 112/158 |
| Positive predictive value | 16 | 8/49 | 34 | 24/70 |
| Negative predictive value | 95 | 82/86 | 97 | 112/116 |

[a]Only first biopsies included in analysis and biopsies showing canoer excluded.

than the one where the carcinoma was localized. The bronchoscopy data, the results of the histologic examination and the performance of conventional bronchoscopy and LIFE in detecting preinvasive lesions are shown in Tables III and V. The relative sensitivity of conventional bronchoscopy combined with LIFE vs conventional bronchoscopy alone was 1.14 (95% CI 0.89–1.48). The results are comparable to those of all bronchoscopies. There was a trend that conventional bronchoscopy combined with LIFE was more sensitive in detecting preinvasive changes in the bronchial mucosa than conventional bronchoscopy alone. However, at cost of a lower sensitivity and positive predictive value. In Table VI the performance of LIFE in detecting preinvasive lesions in the first half of the patient group is compared with the results in the second half. As can be seen from the table there seems to be a learning effect using the LIFE system. Sensitivity, specificity, positive predictive value and negative predictive value all were higher in the second half of the patient group.

### DISCUSSION

Finding more early stage cancer lesions in the central airways which can be treated with curative intent using currently available treatment modalities such as photodynamic therapy or electrocautery may improve the poor prognosis of patients with lung cancer [9–14]. Efforts have been made in applying conventional fiberoptic bronchoscopy to detect and localize early stage lung cancer [16–18]. Even in patients with positive sputum cytology repeated bronchoscopic examinations were necessary and this was despite the fact that only a proportion of the lesions were preinvasive. Therefore the routine use of conventional fiberoptic bronchoscopy seems impractical for screening high risk individuals among the high numbers of smokers with COPD [19], those with a previous history of curatively treated head and neck cancer [20] and those at risk for multiple or subsequent lung cancers [21]. The LIFE system has been developed to detect and localize preinvasive lesions [4,5]. In 53 patients with known or suspected bronchogenic carcinoma and 41 volunteers, the sensitivity of conventional bronchoscopy in detecting moderate/severe dysplasia and carcinoma *in situ* was 48.4% with a specificity of 94%. With fluorescence imaging the sensitivity was 72.5% with the same specificity [4]. In another study by Lam *et al.* the proportion of biopsy proven moderate/severe dysplasia, and carcinoma *in situ* detected by conventional bronchoscopy alone was 38.5% and 40%, respectively. With addition of LIFE examination, the detection rate was 73.1% and 91.4%, respectively. The specificity of conventional bronchoscopy was 91.1%. With fluorescence bronchoscopy, the specificity was 86.7% [5]. In the present study the sensitivities of the LIFE system in detecting moderate/severe dysplasia and carcinoma *in situ* were 80%. When LIFE was added to conventional bronchoscopy the sensitivities improved to 85%. These results are comparable to those mentioned above. In recent studies sensitivities of 90% and 67% in detecting (pre)invasive lesions were reported [22,23]. However, the specificities when using the LIFE system were lower in the present study (60–69%). This resulted in low positive predictive values for detection of preinvasive lesions when LIFE was added to conventional bronchoscopy (23% and 27%). However, in the only other study reporting a positive predictive value this was found to be 23% [23]. The negative predictive values when LIFE was added to conventional bronchoscopy were very high (97%) in the present study. Lam *et al.* recently reported a negative predictive value of 89% [23]. The sensitivities of conventional bronchoscopy were higher in the present study (59% and 70%), with similar specificities. Yokomise *et al.* also found a high sensitivity (65%) of conventional bronchoscopy. However, biopsies showing cancer were included in their analysis [22]. Dysplasia and carcinoma *in situ* are difficult to detect during a standard fiberoptic bronchoscopic examination, because these lesions are superficial and minute in size [21,24]. However, subtle changes may be seen such as increased redness, changes in vascularization, local swelling, relatively thickened mucosa, loss of luster etc. [25], as can be learned by experience while performing careful bronchoscopic examination of the entire tracheobronchial tree. Some preinvasive lesions and even two carcinomas were seen by conventional bronchoscopy but were missed by LIFE. This study therefore confirms that fluorescence bronchoscopy cannot replace conventional bronchoscopy but should be performed in addition to it [23]. Several preinvasive lesions were missed by both conventional bronchoscopy and LIFE. However, histologic examination of these lesions only showed moderate dysplasias. So only the lesions with the lowest degree of preinvasive changes, that were considered to be the interest of using the LIFE system in this study, were missed. The reason of the failure of LIFE to localize some (pre)invasive lesions is not clear. Moreover the meaning of suspicious LIFE findings with negative histology also is unknown. Whether these lesions represent very early changes at the molecular level is unclear. In biopsies taken by others, using the LIFE system, molecular genetic changes such as cumulative gene losses in the progression of preinvasive epithelial lesions have been found [26–28]. The fluorescence pattern of the bronchial mucosa may therefore reflect molecular genetic changes which are beyond the threshold of the macroscopic and microscopic abilities of the bronchoscopist and the pathologist. By using current technique and histological criteria as the golden standard for diagnosing preinvasive lesions, the clinical impact of molecular genetic changes and its relation to tissue autofluorescence remains elusive. A subgroup of patients underwent several LIFE bronchoscopies. To exclude that patient selection bias may influence the results, a separate analysis was performed using only the first bronchoscopy of each patient. The results of this analysis were comparable to those of all the bronchoscopies. The number of preinvasive lesions found with LIFE was higher and the best results were obtained when both modalities were combined. Both analyses showed that specificity and positive predictive were lower when LIFE was used and that a high sensitivity can be obtained by conventional bronchoscopy alone. To study if there was a learning effect in using the LIFE system the results of the first bronchoscopies of the first half of the patient group were compared with the results obtained in the second half. Sensitivity, specificity, positive predictive value and negative predictive value all were higher in the second half of the patient group. This indicates a learning effect in using the LIFE system. We conclude that fluorescence bronchoscopy can be performed safely and that after a learning period it can increase the yield of finding preinvasive neoplastic lesions when used in addition to conventional bronchoscopy.

### *References*

[1] Saccomanno, G., Archer, V.E., Auerbach, O., Saunders, R.P. and Brennan, L.M. Development of carcinoma of the lung as reflected in exfoliated cells. *Cancer* 1974; **33**: 256–270.

[2] Palcic, B., Lam, S., Hung, J. and MacAulay, C. Detection and localization of early lung cancer by imaging techniques. *Chest* 1991; **99**: 742–743.

[3] Hung, J., Lam, S., LeRiche, J.C. and Palcic, B. Autofluorescence of normal and malignant bronchial tissue. *Lasers Surg. Med.* 1991; **11**: 99–105.

[4] Lam, S., MacAulay, C., Hung, J., LeRiche, J., Profio, A.E. and Palcic, B. Detection of dysplasia and carcinoma *in situ* with a lung imaging fluorescence endoscope device. *J. Thorac. Cardiovasc. Surg.* 1993; **105**: 1035–1040.

[5] Lam, S., MacAulay, C., LeRiche, J.C., Ikeda, N. and Palcic, B. Early localization of bronchogenic carcinoma. *Diagn. Ther. Endosc.* 1994; **1**: 75–78.

[6] Watanabe, Y., Shimizu, J., Oda, M., Iwa, T., Takashima, T., Kamimura, R. *et al.* Early hilar lung cancer: Its clinical aspect. *J. Surg. Oncol.* 1991; **48**: 75–80.

[7] Flehinger, B., Kimmel, M. and Melamed, M. The effect of surgical treatment on survival from early lung cancer. Implications for screening. *Chest* 1992; **101**: 1013–1018.

[8] Shimizu, N., Ando, A., Teramoto, S., Moritani, Y. and Nishi, K. Outcome of patients with lung cancer detected via mass screening as compared to those presenting with symptoms. *J. Surg. Oncol.* 1992; **50**: 7–11.

[9] Sutedja, G. and Postmus, P.E. Bronchoscopic treatment of lung tumors. Review article. *Lung Cancer* 1994; **11**: 1–17.

[10] Edell, E.S. and Cortese, D.A. Photodynamic therapy in the management of early superficial squamous cell carcinoma as an alternative to surgical resection. *Chest* 1992; **102**: 1319–1322.

[11] Sutedja, G., Lam, S., LeRiche, J.C. and Postmus, P.E. Response and pattern of failure after photodynamic therapy for intraluminal stage I lung cancer. *J. Bronchol.* 1994; **1**: 295–298.

[12] Furuse, K., Fukuoka, M., Kato, H., Horai, T., Kubota, K., Kodama, N. *et al.* A prospective phase II study on photodynamic therapy with Photofrin II for centrally located early-stage lung cancer. *J. Clin. Oncol.* 1993; **11**: 1852–1857.

[13] Hayata, Y., Kato, H., Furuse, K., Kusunoki, Y., Suzuki, S. and Mimura, S. Photodynamic therapy of 168 early stage cancers of the lung and oesohagus: a japanese multi-centre study. *Laser Med. Sci.* 1996; **11**: 255–259.

[14] Van Boxem, T.J., Venmans, B.J., Schramel, F.M., Van Mourik, J.C., Golding, R.P., Postmus, P.E. *et al.* Radiographically occult lung cancer treated with fibreoptic bronchoscopic electrocautery. A pilot study of a simple and inexpensive technique. *Eur. Resp. J.* 1998; **11**: 169–172.

[15] Venmans, B., Van der Linden, H., Van Boxem, T., Postmus, P., Smit, E. and Sutedja, T. Early detection of pre-invasive lesions in high risk patients. A comparison of conventional fiberoptic and fluorescence bronchoscopy. *J. Bronchol.*, in press.

[16] Saito, Y., Nagamoto, N., Ota, S., Sato, M., Sagawa, M., Kamma, K. *et al.* Results of surgical treatment from roentgenographically occult bronchogenic squamous cell carcinoma. *J. Thorac. Cardiovasc. Surg.* 1992; **104**: 401–407.

[17] Cortese, D.A., Pairolero, P.C., Bergstralh, E.J., Woolner, L.B., Uhlenhopp, M.A., Piehler, J.M. *et al.* Roentgenographically occult lung cancer. A ten-year experience. *J. Thorac. Cardiovasc. Surg.* 1983; **86**: 373–380.

[18] Bechtel, J.J., Kelley, W.R., Petty, T.L., Patz, D.S. and Saccomanno, G. Outcome of 51 patients with roentgenographically occult lung cancer detected by sputum cytologic testing: a community hospital program. *Arch. Intern. Med.* 1994; **154**: 975–980.

[19] Petty, T.L. Lung cancer and chronic obstructive pulmonary disease. *Med. Clin. North Am.* 1996; **80**: 645–655.

[20] Lippman, S.M. and Hong, W.K. Second malignant tumors in head and neck squamous cell carcinoma: The overshadowing threat for patients with early stage disease. *Int. J. Rad. Oncol. Biol. Phys* 1989; **17**: 691–694.

[21] Woolner, L.B., Fontana, R.S. and Cortese, D.A. Roentgenographically occult lung cancer: Pathologic findings and frequency of multicentricity during a 10-year period. *Mayo Clin. Proc.* 1984; **59**: 453–466.

[22] Yokomise, H., Yanagihara, K., Fukuse, T., Hirata, T., Ike, O., Mizuno, H. *et al.* Clinical experience with lung-imaging fluorescence endoscope (LIFE) in patients with lung cancer. *J. Bronchol.* 1997; **4**: 205–208.

[23] Lam, S., Kennedy, T., Unger, M., Miller, Y.E., Gelmont, D., Rusch, V. *et al.* Localization of bronchial intraepithelial neoplastic lesions by fluorescence bronchoscopy. *Chest* 1998; **113**: 696–702.

[24] Usuda, K., Saito, Y., Nagamoto, N., Sato, M., Sagawa, M., Kanma, K. *et al.* Relation between bronchoscopic findings and tumor size of roentgenographically occult bronchogenic squamous cell carcinoma. *J. Thorac. Cardiovasc. Surg.* 1993; **106**: 1098–1103.

[25] Kato, H. and Horai, T. *A Colour Atlas of Endoscopic Diagnosis in Early Stage Lung Cancer*. Wolfe Publishing Tokyo, 1992; p. 15.

[26] Mitsudomi, T., Lam, S., Shirakusa, T. and Gazdar, A.F. Detection and sequencing of p53 gene mutation in bronchial biopsy samples in patients with lung cancer. *Chest* 1993; **104**: 362–365.

[27] Thiberville, L., Payne, P., Vielkinds, J., LeRiche, J., Horsman, D., Nouvet, G. *et al.* Evidence of cumulative gene losses with progression of premalignant epithelial lesions to carcinoma of the bronchus. *Cancer Res.* 1995; **55**: 5135–5139.

[28] Wistuba, I.I., Lam, S., Behrens, C., Virmani, A.K., Fong, K.M., LeRiche, J. *et al.* Molecular damage in the bronchial epithelium of current and former smokers. *J. Natl. Cancer Inst.* 1997; **89**: 1366–1373.

